# A Probiotic *Bacillus velezensis* Consortium Exhibits Superior Efficacy over Two Alternative Probiotics in Suppressing Swine Pathogens and Modulating Intestinal Barrier Function and Immune Responses In Vitro

**DOI:** 10.3390/microorganisms14010249

**Published:** 2026-01-21

**Authors:** Josh Walker, Katrine Bie Larsen, Steffen Yde Bak, Niels Cristensen, Nicolas Chubbs, Weiqing Zeng, Adrian Schwarzenberg, Chong Shen

**Affiliations:** 1 Direct-Fed Microbials Lab, R&D, Health & Biosciences, International Flavors & Fragrance (IFF), Nutrition Biosciences USA 1, LLC, 200 Powder Mill Road, Experimental Station—E361, Wilmington, DE 19803, USA; joshua.walker@iff.com (J.W.); nicholas.chubbs@iff.com (N.C.); weiqing.zeng@iff.com (W.Z.); 2 Gut Immunology Lab, R&D, Health & Biosciences, International Flavors & Fragrance (IFF), Edwin Rahrs Vej 38, 8220 Brabrand, Denmark; katrinebie.larsen@iff.com; 3 Enabling Technologies, R&D, Health & Biosciences, International Flavors & Fragrance (IFF), Edwin Rahrs Vej 38, 8220 Brabrand, Denmark; steffen.yde.bak@iff.com (S.Y.B.); niels.christensen@iff.com (N.C.); adrian.schwarzenberg@iff.com (A.S.)

**Keywords:** *Bacillus velezensis*, pathogenic bacteria, intestinal health, porcine intestinal epithelial cells, probiotic, swine

## Abstract

Despite increasing interest in probiotics as antibiotic alternatives in swine production, few studies have directly compared the functional efficacy of different commercial probiotic formulations under controlled conditions. We conducted an in vitro study using porcine intestinal epithelial (IPEC-J2) and macrophage-like (3D4/21) cell models to compare the efficacy of three commercial probiotic consortia (C1: three strains of *Bacillus velezensis*; C2: *B. licheniformis* + *B. subtilis*; C3: *Clostridium butyricum*). Treatments were evaluated for their ability to inhibit pathogenic *Escherichia coli*, *Clostridium perfringens*, and *Salmonella* spp., enhance epithelial barrier integrity, and modulate immune responses. Experimental endpoints included pathogen inhibition assays, adhesion to IPEC-J2 cells, transepithelial electrical resistance (TEER), tight junction protein expression, and cytokine profiling via RT-qPCR and proteomics. Data were analyzed using the Kruskal–Wallis test with false discovery rate (FDR) control at 5%. C1 cell-free supernatant (CFS) strongly inhibited pathogen growth (84.8 ± 5.3% inhibition of ETEC F4^+^F18^−^ vs. medium control; *p* < 0.05), whereas C2 had no effect, and C3 inhibited only one isolate. The coculture of IPEC-J2 cells with C1 CFS increased the expression of TJ proteins ZO-1, MUC13, and MUC20 (+12.9–46.6% vs. control; *p* < 0.001) and anti-inflammatory TGF-β; reduced pro-inflammatory IL-6 in LPS-stimulated 3D4/21 cells. In comparison, C2 and C3 showed minimal impact on epithelial barrier integrity and immune modulation, as indicated by negligible changes in TEER values, tight junction protein expression (ZO-1, MUC13, MUC20), and cytokine profiles relative to the control. In conclusion, C1 demonstrated greater in vitro efficacy than C2 (*B. licheniformis* + *B. subtilis*) and C3 (*Clostridium butyricum*), including pathogen inhibition assays, epithelial adhesion, TEER measurements, and cytokine modulation, suggesting its potential as a leading candidate for functional probiotic applications.

## 1. Introduction

Post-weaning diarrhea (PWD) remains a challenge in swine production, leading to substantial economic losses through impaired growth performance, increased mortality, and overall inefficiencies [1]. The primary pathogenic bacteria responsible for PWD are enterotoxigenic *Escherichia coli* (ETEC), *Clostridium perfringens*, and various *Salmonella* species [2,3]. In the case of ETEC, the bacterium adheres to epithelial receptors via fimbrial adhesins (F4/K88, F18) and secretes heat-labile (LT) and heat-stable (STa/STb) enterotoxins, which elevate intracellular cAMP and cGMP. This disrupts ion transport by stimulating chloride secretion and inhibiting sodium absorption, creating an osmotic imbalance that drives water into the lumen and results in secretory diarrhea. Infection also compromises tight junction integrity, increases intestinal permeability, and triggers inflammatory cytokine release, further exacerbating fluid loss and epithelial damage. Together, these processes explain the pathogenesis of PWD in weaned piglets [4,5]. Meanwhile, *C. perfringens* is an opportunistic pathogen that can over-proliferate under conditions of stress, dietary change, and pathogen infection after weaning [3]. An increasing demand for antibiotic alternatives in farmed animals has driven research interest in probiotics for their capacity to enhance intestinal health and reduce infections. Commonly employed probiotic species originate primarily from the genera *Lactobacillus*, *Bifidobacterium*, *Bacillus*, *Enterococcus*, and *Streptococcus*, in addition to yeasts from the genus *Saccharomyces* [6]. The addition of probiotics to piglet diets has been shown to improve growth performance, reduce mortality, reduce the prevalence of *E. coli* in feces and the occurrence of diarrhea, and increase the production of short-chain fatty acids (SCFAs) in the cecum, which are utilized by beneficial bacteria for growth and colonization in the host [7,8,9].

*Bacillus*-based probiotics have been shown to promote a healthy gastrointestinal microbiota and enhance growth in swine [10,11]. These effects are thought to be mediated through several mechanisms, including (1) secreting hydrolytic enzymes that can improve nutrient digestibility and utilization, such as glycosidases [12], protease [13], mannanase [14], and lipase [15]; (2) enhancing intestinal health and the microbiome via improved gut barrier integrity, beneficial modulation of the intestinal microflora by competitive exclusion, and the antagonism of pathogens; (3) producing bacteriocins that inhibit bacteria and neutralize enterotoxins; and (4) stimulating the immune system [16,17,18]. In light of these established mechanisms of Bacillus-based probiotics, the present study was designed to interrogate four mechanistic axes in vitro. First, pathogen inhibition assays quantify direct antagonism; second, IPEC-J2 adhesion assays assess epithelial engagement that underlies colonization resistance and receptor-site competition; third, barrier integrity is evaluated via transepithelial electrical resistance (TEER) and RT-qPCR of tight junction and mucin transcripts (ZO-1, MUC13, MUC20), providing targeted readouts of epithelial reinforcement; and finally, untargeted proteomics in IPEC-J2 and porcine macrophage-like 3D4/21 cells profiles cytokines and signaling mediators to resolve immunomodulatory effects, including attenuating pro-inflammatory markers and augmenting anti-inflammatory pathways. Together, these endpoints operationalize prior knowledge into a coherent experimental framework and enable direct functional comparison among the three commercial formulations.

Although probiotics are increasingly explored as alternatives to antibiotics in swine production, comparative, controlled studies directly evaluating the functional efficacy of different commercial formulations are scarce [6,19,20,21,22]. Existing research largely emphasizes growth performance outcomes rather than elucidating mechanisms of pathogen suppression and immune modulation, which creates a gap that limits informed selection of probiotics for optimal intestinal health and disease prevention [6,19,20,21,22,23,24,25,26,27]. In this study, we investigated a commercially available probiotic consortium comprising three strains of *B. velezensis* (called C1) and compared its functional efficacy in vitro with two other commercially available swine probiotics: a two-strain consortium of *B. licheniformis* and *B. subtilis* (C2) and a single-strain probiotic comprising *C. butyricum* (C3). The three probiotics were chosen for commercial relevance, distinct microbial compositions, and documented safety profiles, enabling a meaningful comparative evaluation of functional mechanisms [19,21,22,28,29]. Across swine studies, multi-strain probiotic consortia generally deliver broader and more consistent benefits for weaned piglets than single strains, although the superiority is indication- and strain-specific. A live three-strain blend (*Lactobacillus plantarum*, *Streptococcus thermophilus*, *Bacillus subtilis*) improved the feed conversion ratio, increased the villus height, reduced the crypt depth, and shifted fecal microbiota toward beneficial taxa versus basal diet or inanimate preparations—effects consistent with multiplex mechanisms that single strains seldom reproduce in comparable nursery contexts [30]. Multi-omics analyses of multi-strain lactobacilli in piglets showed enriched commensal *Lactobacillus*, decreased *Clostridium*, and increased acetate and amino acids linked to energy metabolism and tight-junction support, illustrating consortium-level functional breadth beyond a single strain’s metabolic profile [31]. Trials using multispecies blends (e.g., *B. subtilis*, *B. coagulans*, *B. licheniformis*, and *Clostridium butyricum*) further reported favorable *Lactobacillaceae* dominance, feed efficiency, and anti-inflammatory cytokine patterns compared with antibiotic controls, supporting the ecological reach of consortia in the nursery phase [32]. Swine-focused reviews concur that mixtures spanning *Lactobacillus*, *Bacillus*, *Bifidobacterium*, *Enterococcus*, and *Saccharomyces* often outperform single strains through additive/synergistic actions (pathogen exclusion, SCFA production, immune modulation), while cautioning that outcomes remain strain- and endpoint-dependent [33,34]. Therefore, a three-strain consortium designed for complementary colonization resistance, barrier reinforcement, and metabolic support is more likely to outperform two- or single-strain formulations in post-weaning pigs. The C1 consortium has previously been shown in poultry to enhance gut microflora composition, improve gut barrier integrity, and improve growth performance [28,29,35], whilst in swine, C1 has been shown to improve growth performance and enhance the fecal microbiome [19,20]. In vitro, the individual strains comprising C1 have been shown to inhibit poultry pathogens [36] and swine pathogens [18].

Taken together, we hypothesize that the combined effect of the multi-strain *Bacillus velezensis* consortium (C1) demonstrates superior efficacy compared with two other commercial probiotics (C2 and C3) in inhibiting swine pathogens and enhancing intestinal barrier integrity and immune function in vitro. The comparison focused on the capacity of the probiotics to suppress the growth of swine pathogens, improve intestinal epithelial integrity, and enhance the immune response, using porcine intestinal epithelial and monocytic cell models. The goal was to further elucidate the mechanistic basis of the probiotic action of C1 and to assess its performance relative to other commercial probiotics in vitro.

## 2. Materials and Methods

### 2.1. Reagents and Materials

All cell culture media, equipment, and reagents were purchased from Thermo Fisher Scientific (Roskilde, Denmark) unless otherwise stated.

### 2.2. Cell Line, Bacterial Strains, Culture Conditions, and Preparation of Cell-Free Supernatant

The porcine IPEC-J2 cell line was purchased from DSMZ (Braunschweig, Germany) and cultured in Dulbecco’s Modified Eagle Medium, high glucose, GlutaMAX™ Supplement (DMEM), supplemented with 20% fetal bovine serum (FBS) and 1% penicillin/streptomycin (P/S; 100 units (U)/mL of penicillin and 100 µg/mL of streptomycin). The porcine 3D4/21 monocytic cell line was purchased from LGC-ATCC (Hamburg, Germany) and cultured in RPMI 1640 medium containing 2 mM L-glutamine supplemented with 10% fetal bovine serum (FBS) and 1% P/S. Both cell lines were cultured, and the assays were performed at 37 °C in an atmosphere of 5% CO_2_.

The probiotic strains used are listed in Table 1. C1 contained an equal-part combination of *B. velezensis* strains LSSA01, 15AP4, and 2084, which together comprise Enviva^®^ Pro. C1 was manufactured and supplied by Danisco Animal Nutrition & Health, IFF, Oegstgeest, The Netherlands. LSSA01 and 15AP4 were isolated from turkey litter by Agtech Products (Agtech, Inc., Manhattan, KS, USA) in 2002 and 2000, respectively. 2084 is a non-proprietary commercial strain from Novonesis (Bagsværd, Denmark) and Microbial Discovery Group (Oak Creek, WI, USA). C1 is produced through controlled fermentation of selected Bacillus velezensis strains under optimized conditions to maximize sporulation. After fermentation, spores are harvested, concentrated, and stabilized via spray-drying or lyophilization to preserve viability and thermal resistance. The final formulation is standardized to high spore density (≥2.5 × 10^9^ CFU·g^−1^) and demonstrates long-term stability and tolerance to commercial feed pelleting processes. C2 contained equal parts of *B. licheniformis* and *B. subtilis* strains, which together comprise Bioplus^®^ 2B, and was purchased from Novonesis, Kalundborg, Denmark. C3 comprised a single strain of *Clostridium butyricum* (Miya-Gold^®^) and was purchased from Huvepharma, Sofia, Bulgaria. The CC1 [*B. amyloliquefaciens* (*B. velezensis*) *DSM 7*] and CC3 (*B. velezensis* strain 27) strains were IFF in-house strains obtained from the Danisco Global Culture Collection (DGCC; Niebüll, Germany) and used as control strains. The CC2A and CC2B strains were type strains *B. licheniformis DSM 13* and *B. subtilis DSM 10*, respectively, obtained from DSMZ (Braunschweig, Germany) and used as controls. The ETEC, *C. perfringens*, and *Salmonella* isolates were obtained from the Danisco Global Culture Collection (DGCC; Niebüll, Germany). Their identification and genetic characterization are given in Supplementary Table S1.

Unless otherwise noted, *Bacillus* isolates were grown on Tryptic Soy Agar (TSA) or in Tryptic Soy Broth (TSB), both at 32 °C under aerobic conditions. The *C. butyricum* (probiotic) strain was grown on brain heart infusion agar (BHI) or in BHI Broth containing 0.5 g/L of Yeast Extract and 0.05 g/L of L-Cysteine (BHIB + YE + LC; Teknova #B9970), at 37 °C under anaerobic conditions (using Oxoid AnaeroGen sachets).

Cell-free supernatant (CFS) from each probiotic strain was prepared from glycerol freezer stocks by streaking on agar and incubating overnight. Colonies were picked and thoroughly suspended in the appropriate liquid culture medium (broth) in a glass-bottomed flask, and large particles were allowed to settle to the bottom. The suspension at the top of the flask was then aspirated into a new container. The optical density (OD) of the suspension was measured at 600 nm (OD600). The volume of suspension required to produce an OD600 of 1, corresponding to 50 µL of pure culture medium, was calculated according to the following formula:

x volume of suspension required = 50 μL/(ODx − ODy)

where

ODx = OD600 of cell suspension;
ODy = OD600 of culture media alone.

This formula assumes that OD scales linearly with dilution. Only values of less than 1.5 were accepted for ODx; otherwise, the suspension was diluted with additional culture medium (broth) and measured again. The calculated volume of suspension required was used to inoculate a 125 mL flat-bottomed flask containing 25 mL of the appropriate broth. Optical density was measured using a Synergy MX Microplate Reader (BioTek Instruments, Inc., Highland Park, VT, USA). Flasks containing bacterial suspension and broth were incubated with shaking at 21.0× *g* for 6 h. The OD600 of the flask contents was then measured, and the volume required to generate an OD600 value of 1 (corresponding to 10 μL of pure culture medium) was calculated in a similar way to the above formula, but replacing 50 μL with 10 μL. The calculated volume of the suspension was used to inoculate a new 250 mL flat-bottomed flask containing 50 mL of the appropriate broth. This flask was incubated with shaking at 250 rpm for 18 h. The bacterial cultures were then centrifuged at 5000× *g* for 5 min, and the supernatant was sterilized by filtering through a 0.2 µM vacuum filter (Thermo Scientific Nalgene Filter Unit, Wilmington, DE, USA, 500 mL, 0.2 μm aPES Membrane, 75 mm diameter, #566-0020) to obtain CFS. The individual CFS was then combined according to the proportion of the commercial probiotic product and was stored at −20 °C until further use.

**Table 1:** Probiotic and control strains and sources.

Code		Strain Designation	Strain Source
Probiotic Consortium	Individual Strains		
C1	C1A	*B. velezensis*, LSSA01	Enviva^®^ Pro, IFF
	C1B	*B. velezensis*, 15AP4	Enviva^®^ Pro, IFF
	C1C	*B. velezensis*, 2084	Enviva^®^ Pro, IFF
C2	C2A	*B. licheniformis*, DSM 5749	Bioplus^®^ 2B, Novonesis
	C2B	*B. subtilis*, DSM 5750	Bioplus^®^ 2B, Novonesis
C3	C3	*Clostridium butyricum* MIYAIRI 588	Miya-Gold^®^, Huvepharma
Control Strains			
CC1	CC1	*B. amyloliquefaciens* (*B. velezensis*), DSM 7	Type strain, DSMZ
CC2	CC2A	*B. licheniformis*, DSM 13	Type strain, DSMZ
	CC2B	*B. subtilis*, DSM 10	Type strain, DSMZ
CC3	CC3	*B. velezensis*, 27	IFF in-house strain, Danisco Global Culture Collection

### 2.3. Pathogen Inhibition Assay

In the pathogen inhibition assay, we tested the ability of CFS derived from each probiotic (C1, C2, and C3, with CFS from each strain, and combined evenly) to inhibit the growth of ETEC, *C. perfringens*, and *Salmonella* spp. isolates. Details of the isolates tested are listed in Supplementary Table S1, all originating from pig farms, and their individual sources are described in Rasmussen et al. [18]. Unless otherwise stated, the ETEC and *Salmonella* isolates were grown on TSA or in TSB, both at 37 °C under aerobic conditions, and the *C. perfringens* isolates were grown on BHI agar or in BHI Broth containing 0.5 g/L of Yeast Extract and 0.05 g/L of L-Cysteine (BHIB + YE + LC; Teknova #B9970), at 37 °C under anaerobic conditions (using Oxoid AnaeroGen sachets). Isolates were revived from freezer stocks by streaking on agar and incubated overnight. Colonies were picked from agar and resuspended in duplicate into 200 μL of the appropriate broth in a microplate (Corning #3474), which was then incubated for 4 h at 37 °C with shaking at 200 rpm. This inoculum culture was used to inoculate wells in duplicate microplates containing broth combined with CFS prepared from the probiotic strains listed in Table 1. Wells containing inoculum culture without CFS served as the control. To test the effect of probiotic CFS on the isolates of ETEC and *Salmonella* spp., 20% *v*/*v* of CFS and 1% *v*/*v* of inoculum culture were used, and to test its effect on *C. perfringens*, 5% *v*/*v* of CFS and 4% *v*/*v* of inoculum culture were used. The total volume per well in each case was 200 μL. After inoculation, the microplates were incubated at 37 °C for 16 h with shaking at 100 rpm, after which the absorbance of the contents of each well was then read at an OD of 600 nm, and the percentage inhibition of pathogen isolate growth was calculated according to the following equation:

% inhibition = [(1 − (ODa − OD0)/(ODb − OD0))] × 100

where

ODa = OD600 of media containing pathogen inoculum and probiotic CFS (challenge well);
OD0 = OD600 of media without inoculum or probiotic CFS (media control);
ODb = OD600 of media containing pathogen inoculum but without probiotic CFS (unchallenged control).

Experiments were performed three times with three replicates per experiment.

As a quality control (QC) measure, absorbance data from any isolates that did not grow sufficiently (defined as OD600 < 0.5 for the unchallenged control) were excluded from data analysis. Outliers were identified by calculating the interquartile range (IQR); values greater than the third quartile plus 1.5 times the IQR or less than the first quartile minus 1.5 times the IQR were considered outliers and removed from the dataset. After these QC procedures, the average percentage inhibition was determined for 17 ETEC isolates, 11 *C. perfringens* isolates, and 7 *Salmonella* isolates.

### 2.4. Probiotic IPEC-J2 Cell Adhesion Assay

The adhesion assay was performed as described in [18]. In brief, IPEC-J2 cells in culture medium were seeded in 96-well cell culture plates at a density of 2 × 10^4^ cells/well, in a total volume of 0.2 mL, and the cells were grown for two days until 100% confluency. The probiotic *Bacillus* strains comprising C1, C2, and C3 (as listed in Table 1) were grown individually in TSB at 37 °C under aerobic conditions, and *C. butyricum* was grown in BHI at 37 °C under anaerobic conditions. After 48 h, the OD600 of the bacterial cultures was measured in each well, and the concentration was adjusted to an OD of 1 by adding culture medium. A 30 μL sample of the bacterial culture was then added directly onto the IPEC-J2 cells in each well, and the cells were cocultured for 30 min at 37 °C. The generated cell monolayer was washed five times with phosphate-buffered saline (PBS), and the cells were lysed with a cold 0.1% Triton X-100 solution. The lysates were serially diluted (10-fold) in PBS and plated onto TSA or BHI agar (as appropriate). Plates were cultured at 37 °C for 24 h, and colonies were then counted to determine the number of adhered bacterial CFU (CFU_adhered_). To determine the number of CFU loaded (CFU_loaded_), samples of the bacterial suspensions were separately serially diluted prior to their addition to the IPEC-J2 cells and plated onto TSA or BHI plates for colony counting, as before. The percentage adhesion of bacteria to the IPEC-J2 cells was calculated according to the following formula:

Cell adhesion (%) = [(CFU_adhered_)/(CFU_loaded_)] × 100

The data are means from two experiments with five replicates in total.

### 2.5. Trans-Epithelial Electrical Resistance (TEER) Assay

The TEER assay was performed as described by Shen et al. [37]. Porcine epithelial cells (IPEC-J2) were differentiated on permeable filters (Thincert Pore 0.4 µm, Greiner Bio-one). Briefly, on day 1, cells were seeded at a density of 5.0 × 10^4^ cells/well on cell culture inserts in complete cultivation medium and differentiated for 21 days. On the 4th day, the cells were moved to asymmetric serum conditions by replacing the medium on the apical side with serum-free cultivation medium and continuing to use complete medium on the basal side of the inserts. Cells continued to differentiate, and the culture media were refreshed every three to four days. On day 20, the TEER of each insert was measured (Value _before_), and only inserts with values >20,000 Ω/cm^2^ were selected for inclusion in the assay, as a quality control measure. For the assay, CFS was added to the apical compartment and the inserts were cultured overnight; on the following day (day 21), lipopolysaccharide (LPS, 100 ng/mL) was added for 4 h, and TEER was measured again (Value_after_). The change in TEER after the CFS was added is expressed as a percentage, according to the following formula:

Change in TEER (%) = (Value_after_/Value_before_) × 100

The experiments were performed in triplicate on two separate occasions, resulting in a total of six replicates for each experimental condition.

### 2.6. Real-Time Quantitative Polymerase Chain Reaction (RT-qPCR) Analysis of IPEC-J2 Gene Expression Without or with Probiotic CFS Pre-Treatment

Porcine IPEC-J2 cells were seeded in 96-well cell culture plates at a density of 2 × 10^4^ cells/mL in Dulbecco’s Modified Eagle Medium (DMEM), with a total volume of 200 µL per well, and were differentiated for 2 days at 37 °C until confluency. On day 2, a CFS probiotic or bacterial control strain was added to reach a final concentration equivalent to that produced by 1 × 10^7^ CFU/mL per well, and the cells were incubated for 6 h at 37 °C in an atmosphere of 5% CO_2_. Cells (IPEC-J2) in culture medium with 10% TSB or 10% BHI media (as appropriate according to the respective active treatment) served as the control. After incubation, the cells were washed with PBS and lysed in 200 µL of RLT buffer (Qiagen, Hilden, Germany) for 20 min, before adding proteinase K (Merck Darmstadt, Germany) to reach a final concentration of 100 µg/mL. The lysate was then harvested, and the expression of selected cytokines and tight junction (TJ) proteins was analyzed by RT-qPCR. RNA extraction and RT-qPCR were performed by Biotest (Trige, Denmark) according to the method described in [18], using the TaqMan assays listed in Supplementary Table S2. The percentage changes in the expression of cytokines IL-6, TGF-β, and tight junction proteins zonula occludens-1 (ZO-1), mucin 13 (MUC13), and mucin 20 (MUC20) were determined as described in [18]. Prior to analysis, data were normalized to two sets of housekeeping genes (listed in Supplementary Table S2) according to the following formula:

Gene expression value = 2^−(Ct sample−Ct housekeeping)^ × 10^3^

where Ct is the cycle threshold of the target gene.

The fold-change in gene expression in the probiotic CFS-treated wells compared to the control wells without probiotic CFS was then calculated according to the following formula:

Fold-change in gene expression (%) = [Gene expression value with probiotic CFS/gene expression value in medium control CFS)] × 100

Each experimental condition contains 8 replicates.

### 2.7. Proteomics Analysis of IPEC-J2 and 3D4/21 Cell Protein Expression Without or with Probiotic CFS Pre-Treatment

IPEC-J2 cells were seeded in 96-well cell culture plates until confluency, as described for the cell adhesion assay. The culture medium was then refreshed, and probiotic CFS (or control strain, as appropriate) was added to reach a final concentration equivalent to that produced by 1 × 10^7^ CFU/mL. The cells were further incubated overnight at 37 °C in an atmosphere of 5% CO_2_. Cells in culture medium without added probiotic CFS served as the control.

3D4/21 cells were cocultured with probiotic CFS for 2 h at 37 °C in an atmosphere of 5% CO_2_ and then stimulated with or without LPS (100 ng/mL) and further cultured overnight. Cells were then washed with PBS, and the cell pellets were lysed using 5% SDS solution. Lysates were collected, and the expression of cytokines, chemokines, and transcription factors was quantified by proteomics as described in [38], with a few modifications. Nano LC-MS/MS was performed using a Vanquish Neo nano UHPLC system (Thermo Scientific, Waltham, MA, USA) interfaced to a Q-Exactive-HF Mass Spectrometer (Thermo Scientific), and this system was operated using a trap/elute two-column system. Peptides were trapped on a 20 mm NanoViper Trap Column (Acclaim™ PepMap™ 100 C18, 3 µm particle size, i.d. 0.075 mm) and separated on a Waters nanoEase M/Z Peptide BEH C18: 10 cm × 150 μm − 1.7 μm. Separation was performed at a flow rate of 2000 nl/min using a 70 min gradient. Data were recorded in data-dependent MS/MS mode using HCD fragmentation. Raw data were imported into Proteome Discoverer (Version 3.0, Thermo Scientific) and searched against a UniProt *Sus Scrofa* FASTA database, using an internal Mascot server. Each experimental condition contains 8 replicates.

### 2.8. Statistical Analysis

The study was a multi-assay trial with a total of 17 treatments (probiotics and controls), both as individual strains and probiotic consortia, as outlined in Table 1. Pathogen inhibition assay is based on 7 treatments (6 consortia and control). Bacterial cell adhesion assay is based on 10 treatments (individual bacterial strain). TEER, RT-PCR and proteomics assays are based on 7 treatments (6 consortia and control). The experimental unit was genuine biological replicates (*n* = 5 for probiotics IPEC-J2 cell adhesion assay; *n* = 6 for TEER assay, and *n* = 8 for all the others) for all 17 treatments and for all assay endpoint manifestations. Of interest was a comparison of probiotics vs. controls within each assay for the respective endpoint manifestations.

Data from all assays were analysed by a one-way ANOVA for the different settings of the treatment factor (7-level factor for CFS-related assays and 10-level factor for bacterial cell adhesion assay), using the non-parametric Kruskal–Wallis H test, and the multiple comparisons of interest (probiotics vs. controls) were derived subsequently. The false discovery rate (FDR) for multiple comparisons is controlled at 5% with adjusted significance at the 5% level (*p* < 0.05). All statistical analyses were conducted in GraphPrism Software (version 9).

## 3. Results

### 3.1. Cell-Free Supernatant from Probiotics C1 and C3 Inhibited the Growth of ETEC, C. perfringens, and Salmonella spp. Isolates

**Figure 1:**
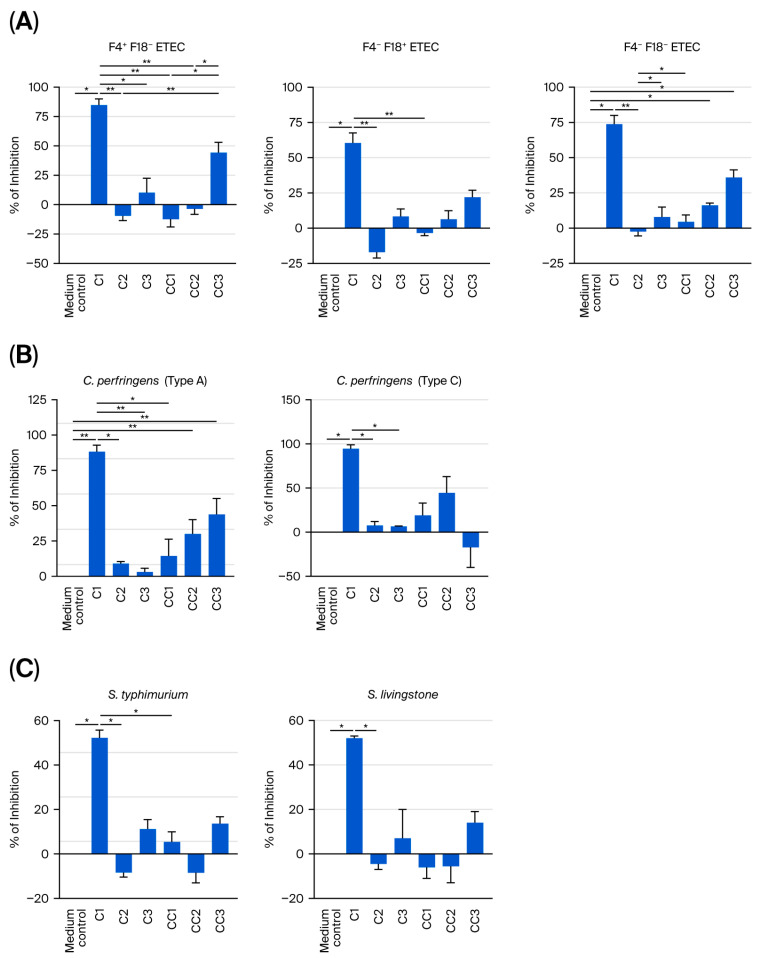
Effect of cell-free supernatant (CFS) from probiotics C1, C2, and C3, their respective control strains, and the medium control on the growth of isolates of ETEC (**A**), *C. perfringens* (**B**), and *Salmonella* spp. (**C**) after 16 h of incubation. Values are shown as means ± standard error (SE). Experiments were performed three times with three replicates per experiment. Pair-wise comparisons to the medium control were performed, and only differences with statistical significance are shown. *: *p* < 0.05; **: *p* < 0.01. *Bacillus* strain identifications are given in Table 1.

Figure 1 shows the inhibitory effect of CFS prepared from each probiotic (C1, C2, and C3), controls (CC1, CC2, and CC3), and the culture medium control (‘medium control’) on the growth of pathogenic isolates of ETEC, *C. perfringens*, and *Salmonella* spp. after 16 h of incubation. The CFS from C1 exhibited a potent inhibitory effect on multiple ETEC phenotypes, whereas CFS from C2 and C3 did not (Figure 1A); the percentage inhibition in the C1 CFS-treated group was higher than that in the medium control for ETEC isolates F4^+^F18^−^ (84.8% ± 5.3% vs. 0.0 ± 0.0%; *p* < 0.05), F4^−^F18^+^ (60.4 ± 7.2% vs. control; *p* < 0.05), and F4^−^F18^−^ (73.8 ± 6.2% vs. control). In addition, CFS from CC2 and CC3 inhibited the growth of ETEC isolate F4^−^F18^−^ compared with the medium control (*p* < 0.05; Figure 1A). Compared with the medium control, CFS from C1 also inhibited the growth of isolates of *C. perfringens* (type A, 88.3% ± 4.5% vs. control, 0.0 ± 0.0%, *p* < 0.05; type C, 94.5% ± 4.5% vs. control, 0.0 ± 0.0%, Figure 1B) and *Salmonella* spp. (*S. typhimurium,* 52.2% ± 3.5% vs. control, 0.0 ± 0.0%, *p* < 0.05; *S. livingstone*, 52.0% ± 1.0% vs. control 0.0 ± 0.0%, *p* < 0.05, Figure 1C). Neither C2 nor C3 CFS inhibited these isolates (individual data listed in Supplementary Table S3).

### 3.2. Probiotic C1 Exhibited Superior Binding Affinity to IPEC-J2 Cells

**Figure 2:**
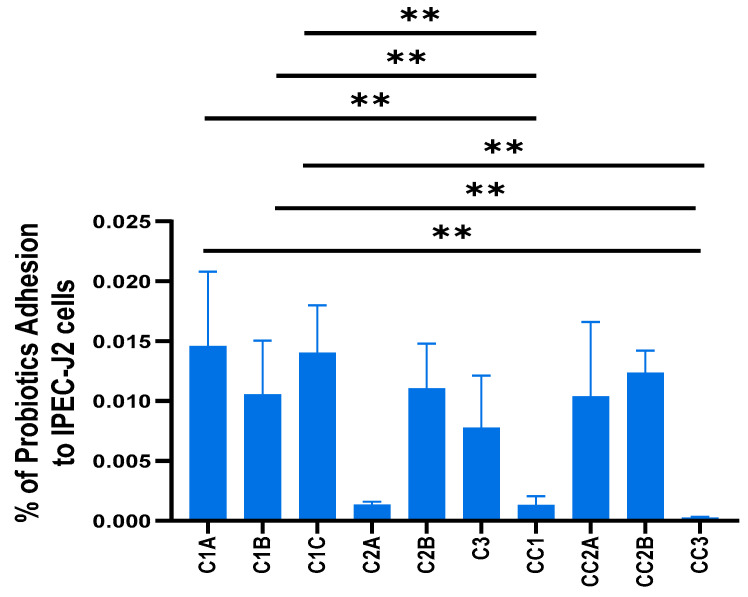
Binding affinity (% cell adhesion) of individual strains (as colony-forming units) of C1 (C1A, C1B, and C1C), C2 (C2A, and C2B), C3, and their respective control strains (CC1, CC2A, CC2B, and CC3) to IPEC-J2 cells, after 30 min of coculture. The data represent the results of two experiments with five replicates in total. Values are shown as means ± standard error (SE). Pair-wise comparisons to CC1, CC2A, CC2B, and CC3 were performed, and only differences with statistical significance are shown. **: *p* < 0.01. *Bacillus* strain identifications are given in Table 1.

The binding affinity of CFU of the individual bacterial strains within each probiotic to IPEC-J2 cells after coculture for 30 min is shown in Figure 2. The three probiotic *Bacillus velezensis* strains within C1 (C1A, C1B, and C1C) exhibited higher binding affinity to IPEC-J2 cells than the *B. velezensis* control strains (C1A: 0.015 ± 0.006%; C1B: 0.011 ± 0.004%; C1C: 0.014 ± 0.004 vs. control strains CC1: 0.001 ± 0.001% and CC3: 0.000 ± 0.000%; *p* < 0.01). By contrast, probiotic C2 strains C2A and C2B and the probiotic strain within C3 exhibited lower binding affinities after coculture (range: 0.001 to 0.011%).

### 3.3. Preservation of Barrier Integrity and Upregulation of Tight Junction Proteins in IPEC-J2 Cells by C1 Cell-Free Supernatant

**Figure 3:**
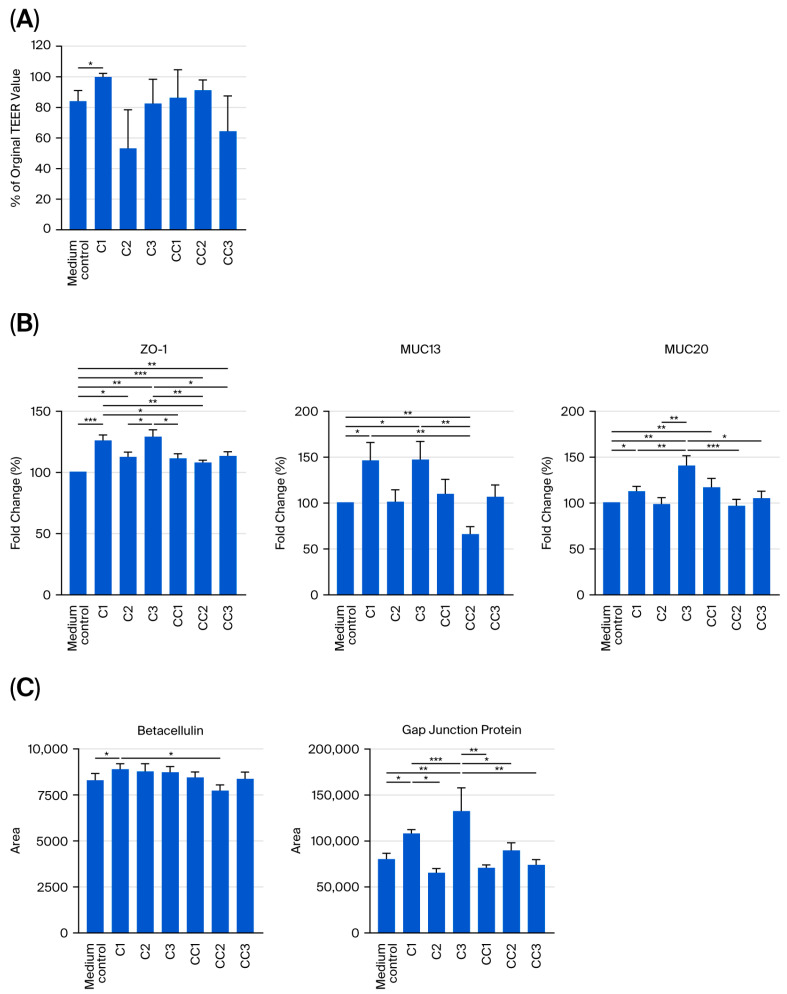
Effect of pre-treatment with cell-free supernatant (CFS) from probiotics C1, C2, and C3, their respective control strains, or the medium control on (**A**) IPEC-J2 cell TEER and (**B**) IPEC-J2 mRNA expression of TJ proteins ZO-1 and mucins, both determined by RT-qPCR, and on (**C**) protein abundance of betacellulin and gap junction protein, determined by proteomics. Values of TEER in (**A**) represent the value obtained after incubation with CFS as a percentage of TEER before incubation with CFS; TJ protein expression in (**B**) is expressed as the percentage change relative to the medium control. All values represent means and associated standard errors (SEs). The data represent the results of 8 replicates in total. Pair-wise comparisons to the medium control were performed, and only differences with statistical significance are shown. *: *p* < 0.05; **: *p* < 0.01; ***: *p* < 0.001. The identity of the probiotic sources is given in Table 1.

The percentage change in the TEER of IPEC-J2 cells after (relative to before) incubation with CFS from probiotics C1, C2, and C3, their respective control strains, or the medium control is shown in Figure 3A. IPEC-J2 cells treated with C1 CFS exhibited no change in TEER after incubation (TEER value after incubation as a percentage of TEER before incubation was 99.9% ± 2.3%). This contrasted with IPEC-J2 cells treated with the medium control, which exhibited a 16.4% reduction in TEER after incubation (to 83.6% ± 7.4%; *p* < 0.05). By contrast, there is no effect observed in the coculture of the C2 (to 53.3% ± 25.2%) or C3 (to 82.6% ± 15.9%) CFS.

The fold-change (%) in the mRNA expression of TJ proteins by IPEC-J2 cells treated with CFS from probiotics C1, C2, and C3 or their respective controls, relative to expression in the medium control, as determined by RT-qPCR, is shown in Figure 3B. CFS from probiotics C1 and C3 significantly increased the expression of ZO-1, MUC13, and MUC20 (fold-change vs. medium control: C1: 126.1% ± 4.5% (*p* < 0.001) +46.6% ± 19.4% (*p* < 0.05) and +112.9% ± 5.3% (*p* < 0.05), respectively). By comparison, C3 showed 129.3% ± 5.6% (*p* < 0.001) for ZO-1, 147.3% ± 19.8 (*p* < 0.01) for MUC13, and 141.0% ± 10.6% (*p* < 0.01) for MUC20. CFS from probiotic C2 had no effect on the expression of these TJ proteins.

The proteomic quantification of structural proteins involved in epithelial cohesion and communication in IPEC-J2 cells treated with either probiotic CFS, their respective control strains, or the medium control is shown in Figure 3C. The abundance of betacellulin and gap junction protein was increased in IPEC-J2 cells treated with C1 CFS compared with the medium control (area: 8456.3 ± 298.5 vs. 8262.4 ± 407.3 and 108,324.7 ± 4169.8 vs. 7955.0 ± 7214.0, for betacellulin and gap junction protein, respectively; *p* < 0.05), whereas there was no significant increase in the abundance of these proteins in cells treated with C2. CFS from C3 increased the abundance of gap junction protein in IPEC-J2 cells (132,560.3 ± 25,302.8 vs. 79,522.0 ± 7214.0 in the medium control; *p* < 0.05).

### 3.4. Anti-Inflammatory Cytokine Expression of IPEC-J2 and 3D4/21 Cells Treated with Probiotic C1 Cell-Free Supernatant

**Figure 4:**
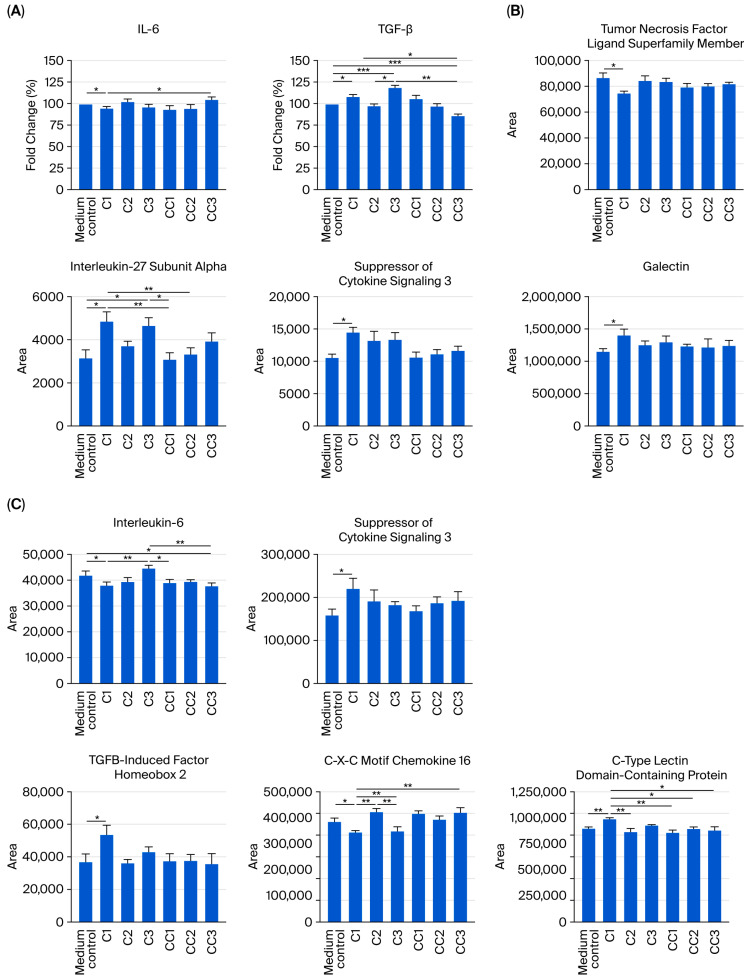
Effect of pre-treatment with cell-free supernatant (CFS) from probiotics C1, C2, and C3, their respective control strains, or a medium control on (**A**) cytokine expression and cell signaling protein abundance in IPEC-J2 cells; (**B**) cell signaling protein abundance in unstimulated 3D4/21 cells; and (**C**) cell signaling protein abundance in LPS-stimulated 3D4/21 cells. CT values in the RT-qPCR assay used to measure gene expression were normalized to two sets of housekeeping genes using the equation: Value = 2^−(Ct sample−Ct housekeeping)^ × 10^3^. The data represent the results of 8 replicates in total. All values represent means and associated standard errors (SEs). Pair-wise comparisons to the medium control were performed, and only differences with statistical significance are shown. *: *p* < 0.05; **: *p* < 0.01; ***: *p* < 0.001. The identity of the probiotic sources is given in Table 1.

The effect of pre-treatment of IPEC-J2 and 3D4/21 cells with CFS from C1, C2, and C3 on cytokine gene expression, as analyzed by RT-qPCR, and on the abundance of expressed proteins, as analyzed by proteomics, is shown in Figure 4. Pre-treatment of IPEC-J2 cells with CFS from C1 significantly reduced the expression of pro-inflammatory IL-6 (fold-change vs. medium control: 94.1% ± 2.5%; *p* < 0.05; Figure 4A) and increased the expression of anti-inflammatory TGF-β (fold-change vs. medium control: 107.6% ± 2.9%; *p* < 0.05; Figure 4A). CFS from C1 consistently increased the abundance of anti-inflammatory IL-27 subunit α in IPEC-J2 cells compared with the medium control (C1: 4839.8 ± 455.3 vs. medium control: 3177.0 ± 357.0; *p* < 0.05) and reduced the abundance of the suppressor of cytokine signaling 3 protein (C1: 14,439.1 ± 820.3 vs. medium control: 10,666 ± 435.0; *p* < 0.05; Figure 4A). Meanwhile, CFS from C3 enhanced the expression of TGF-β (fold-change vs. medium control: 100.8% ± 3.2%, *p* < 0.001) and the abundance of IL-27 subunit α (4641.9 ± 381.8 vs. medium control; *p* < 0.05) but had no significant effect on the expression of IL-6 or the abundance of the suppressor of cytokine signaling 3 protein. Pre-treatment of cells with CFS from probiotic C2 had no effect on the expression of the measured cytokines (Figure 4A).

In unstimulated 3D4/21 cells, the abundance of pro-inflammatory cytokines, a member of the tumor necrosis factor ligand superfamily, was reduced following treatment with CFS from C1, relative to the response in the medium control, as analyzed via proteomics (74,336.8 ± 1919.6 vs. 87,092.0 ± 3306.0, respectively; *p* < 0.05; Figure 4B). In addition, the abundance of galectin, a cytokine that regulates both innate and adaptive immune responses, was increased (1,399,844.0 ± 99,284.7 vs. 1,160,881.0 ± 35,049.0; *p* < 0.05). Treatment of cells with CFS from C2 or C3 had no significant effect on the expression of these cytokines.

In LPS-stimulated 3D4/21 cells that model the acute inflammatory response, treatment with CFS from probiotic C1 led to a pronounced reduction in the abundance of inflammatory cytokines in these cells (Figure 4C); IL-6 abundance in cells was significantly reduced compared with the untreated, LPS-stimulated, medium control (37,869.2 ± 1447.9 vs. 42,110.9 ± 1456.3; *p* < 0.05). Meanwhile, the abundance of anti-inflammatory cytokines was increased compared to the medium control, including the suppressor of cytokine signaling 3 protein (219,667.6 ± 24,966.6 vs. 160,183.7 ± 12,770.8; *p* < 0.05) and TGFB-induced factor homeobox 2 (53,398.4 ± 5986.8 vs. 37,202.4 ± 4548.4; *p* < 0.05), while the abundance of C-X-C motif chemokine 16 was reduced (342,874.9 ± 7845.1 vs. 387,473.9 ± 10,971.4; *p* < 0.05) and that of C-type lectin domain-containing protein was increased (984,581.4 ± 15,180.0 vs. 902,621.7 ± 8747.5; *p* < 0.01). None of these effects were evident with CFS from C2 or C3.

## 4. Discussion

Our results have demonstrated that CFS derived from the combined probiotic strains contained within C1 inhibited the growth of isolates of multiple pathogens from pig farms, including ETEC, *Clostridium perfringens*, and *Salmonella* spp., whereas CFS from probiotics C2 and C3 inhibited only a single ETEC isolate out of all tested isolates. Our previous research on C1 observed growth inhibition of these pathogen isolates by the individual strains when tested separately [18], whereas the present results confirm that the same effect was observed when the strains were tested together (as CFS). It is known from our previous research that the strains within C1 (LSSA01, 15AP4, and 2084) have a relatively high affinity for adhering to porcine gut epithelial (IPEC-J2) cells in vitro [18]. This may be part of the mechanism by which growth inhibition is achieved. A high binding affinity of the strains within C1 (relative to that of the control *Bacillus velezensis* strain) was equally observed in the present study, whereas adhesion of the strains within C2 and C3 to IPEC-J2 cells was generally lower and not different from the control strains. The proposed mechanisms by which probiotic *Bacillus* beneficially modulate the gut microbial composition of piglets involve promoting the growth of beneficial bacteria and suppression of pathogenic bacteria [39]. Our previous research indicated that probiotic *B. velezensis* strains compete with gut pathogens for adhesion sites, thereby inhibiting the attachment and colonization of harmful microbes in the intestinal tract [18]. On the other hand, *Bacillus* spp. are also recognized as carbohydrate fermenters [40], producing substantial amounts of lactic acid, which lowers intestinal pH and creates an anaerobic, acidic environment conducive to the growth of beneficial bacteria such as *Lactobacillus* and *Bifidobacterium* spp., while inhibiting the invasion of pathogens [20,41,42]. In addition, *Bacillus* spp. are capable of secreting a variety of metabolites with antibacterial or protective properties, such as antimicrobial peptides (AMPs) [29] and short-chain fatty acids (SCFAs) [43], which exert antagonistic activity against pathogens and help regulate the balance of the intestinal microbiota. *Bacillus* spp. can also promote the host production of mucins (such as sialomucin and sulfomucin) [24] and enhance mucosal immunity by stimulating secretory immunoglobulin A (SIgA) [25], both of which support the physical barrier against pathogen colonization in the intestine. Any or all of these chemical mediators could explain the inhibitory effect of C1 against isolates of ETEC, *C. perfringens*, and *Salmonella* spp. observed in the present study, the immune modulatory and growth performance effects of C1 that have been noted in previous non-challenge trials [19,20], and the wider health and growth performance benefits of *Bacillus* supplementation observed in ETEC challenge trials in swine [26,27].

The RT-qPCR analyses showed that CFS from both C1 and C3 positively affected IPEC-J2 expression of the TJ protein ZO-1, whilst proteomic analysis further confirmed that all three quantified epithelial cohesion and communication proteins were upregulated by treatment with CFS from C1, whereas only the gap junction protein was upregulated by C3, and none of these proteins were increased by C2. These results suggest a superior effect of C1 over C2 or C3 on barrier integrity under the test conditions and could explain the higher TEER value obtained from C1-treated cells, indicating enhanced epithelial integrity. In addition to these results, pre-treatment of the IPEC-J2 cells with CFS from probiotics C1 and C3 (but not C2) increased the expression of MUC13 (C1 and C3) and MUC20 (C3 only). These transmembrane proteins (mucins) are produced by enterocytes and are situated on the apical surface of the intestinal epithelium, where they participate in important physiological processes and signaling pathways associated with lubrication of the mucosal surface and maintenance of epithelial barrier integrity [44]. Dietary *Bacillus* has previously been shown to upregulate MUC2 (secreted mucin) [24] but not MUC13; our data are the first to indicate that transmembrane mucin may be a contributor to the beneficial effects of *Bacillus* on swine intestinal health and to suggest that MUC13 may play a role in the beneficial effect of C1 on swine intestinal health. As MUC13 was also increased by C3, it may similarly be involved in the beneficial effect of *Clostridium butyricum* in C3.

Immunologically, we observed that CFS derived from C1 suppressed several pro-inflammatory markers in both epithelial (IPEC-J2) and macrophage-like (3D4/21) cells, while enhancing multiple anti-inflammatory markers and cell-signaling molecules, whereas, except for a positive effect of C3 on TGF-β expression in IPEC-J2 cells, CFS from C2 and C3 showed little or no effect. Specifically, CFS from C1 reduced the expression of pro-inflammatory IL-6, increased anti-inflammatory TGF-β in both cell types, and decreased levels of pro-inflammatory tumor necrosis factor ligand superfamily member proteins in macrophage-like cells. These results suggest a multifaceted beneficial immunomodulatory effect of the bacterial strains within C1 on swine immune responses. CFS from C1 also enhanced the expression of C-type lectin domain-containing proteins (CLECs). CFS from C1 upregulated C-type lectin domain-containing receptors (CLECs), a family of pattern-recognition receptors (e.g., Dectin-1/CLEC7A, Dectin-2, Mincle) expressed on myeloid and epithelial cells that bind carbohydrate ligands and signal via Syk/CARD9 to calibrate cytokine production, barrier function, and adaptive priming—processes central to intestinal surveillance [45,46]. Porcine evidence supports functional CLEC signaling: polymorphisms in porcine Dectin-1 (CLEC7A) alter downstream NF-κB activation upon β-glucan ligation, highlighting a role for CLEC7A in swine innate immunity and intestinal homeostasis [47], and β-glucans have been shown to enhance intestinal barrier integrity and modulate immune responses in pigs, further reinforcing the relevance of CLEC-mediated pathways in gut health [48]. These CLEC-linked changes are relevant to current efforts in swine production to reduce post-weaning gut inflammation. This is based on the understanding that inflammation diverts nutrients and energy away from growth toward the immune response. Indeed, it has been shown that even low-grade gut inflammation reduces nutrient absorption, impairs weight gain, and increases feed costs [49]. Thus, the C1-induced upregulation of CLECs—receptors that integrate pathogen recognition with inflammatory signaling—together with probiotic-mediated anti-inflammatory shifts, provides a mechanistic basis for the superior immunomodulatory profile of C1 compared with C2 and C3. Therefore, strategies that reduce inflammation can promote growth and enhance feed efficiency, potentially lowering feed costs. Existing in vivo studies on C1 in swine have indicated an improved feed conversion ratio, reduced mortality, enhanced fecal microbiota composition, and enhanced carcass quality in pigs supplemented with the probiotic consortia [19,20], whilst in vivo studies of C3 in weaned piglets have indicated improved growth performance, intestinal morphology, and beneficial effects on the immune response and microbiome composition [50]. The present results relating to C1 support the existing research in vivo, indicating that it exhibits immunomodulatory properties that improve and balance the immune response, ensuring effective pathogen defense without triggering an excessive inflammatory response.

Some studies have shown that, in addition to beneficial effects from single strains of *Bacillus* spp. administered individually, there can be synergistic effects on piglet growth performance when *Bacillus* spp. are administered in combination with other probiotics or functional substances [25,51]. Our previous study [18] demonstrated that, while not exclusively, each probiotic strain within C1 targets specific functionalities: All three inhibited ETEC isolates, whilst C1B (15AP4) strongly inhibited *C. perfringens* isolates, and C1C (2084) was more effective against *Salmonella* spp. Meanwhile, C1A (LSSA01) exclusively promoted wound-healing, whereas both C1A (LSSA01) and C1C (2084) increased the expression of TJ proteins [18]. The results of the present study confirm these functionalities when the three strains are combined and tested together, indicating their compatibility. The data also indicate a wider spectrum of effects on TJ proteins (including ZO-1, mucins, cadherin, and gap junction protein) when the strains are tested in combination, as C1, compared with the individual strains. Furthermore, effects on multiple anti-inflammatory cytokines were only observed in the present study, not the previous study by Rasmussen et al. [18]; it appears that combining probiotic strains together can result in a wider spectrum of beneficial effects, as previously discussed [52]. The positive interrelationship between strains in C1 could be due to the exchange of different metabolites, which leads to a higher diversity of microflora and a broader efficacy spectrum [52,53].

While C1, C2, and C3 have each demonstrated efficacy in animal trials [19,20,21,22], the present results indicate a substantial difference in their in vitro efficacy under the test conditions, with C1 exhibiting higher efficacy in most measured responses compared with C2 or C3. This finding underscores a key challenge in translating probiotic performance from in vitro (lab-based) to in vivo (live animal) settings. Several important biological and environmental factors in the in vivo setting cannot be replicated in vitro, including interactions between probiotics (and pathogen isolates) and the host microflora, host status (age, gender, breed, and health), delivery format (vegetative cell vs. spores), and dosage and timing of administration. Secondly, probiotic efficacy in vitro appears to be highly strain-specific and context-dependent, as illustrated by the widely varying nature and degree of functional effects reported by genera, species, and strains in in vitro studies [54,55,56,57]. Generalizing results across species or genera can be misleading, and individual strains must be tested to establish efficacy and compatibility prior to their combination. Third, it is important to note that the application dose of probiotics materially influences experimental readouts and real-world efficacy. While C1 efficacy has been validated in piglet trials at 3 × 10^8^ CFU/kg [19,20], C2 bacillus formulations indicate efficacy at 1.3 × 10^9^ CFU/kg in complete feed [21], and C3 Clostridium butyricum is recommended at 2.5 × 10^8^ CFU/kg [22]. These data indicate that under-dosing (below ~10^8^–10^9^ CFU/kg, depending on strain) increases the variability in colonization dynamics, competitive exclusion, immune modulation, and trial reproducibility. Therefore, dose–response characterization should be incorporated into formulation and application strategies to ensure consistent efficacy [39,40]. Lastly, the delivery form of the probiotic administered during in vitro testing should be considered. Xie et al. [55] observed that spore forms (CFU, the cellular state of C1 and C3) demonstrated greater efficacy in immunomodulating the gut microbiota under anaerobic conditions, while vegetative cells (the form used for C2) were more effective in cell-to-cell interactions. To improve the predictive value of in vitro assays for in vivo efficacy, it will be important to incorporate more factors representative of the host gut environment. This could include (1) simulating gastrointestinal conditions (e.g., gastric and intestinal fluids, modeling transit time); (2) co-culturing with gut microbiota by introducing pig fecal microbiota to better mimic native ecological interactions and competitive fitness; and (3) employing a comprehensive biomarker panel, encompassing enzyme production, antimicrobial peptide secretion, and metabolomic analysis to monitor SCFA production, ammonia reduction, and bile acid transformation.

## 5. Conclusions

This is one of the few studies that have directly compared the in vitro efficacy of commercially available probiotics used in swine production. Whilst caution should be applied in translating the results to the in vivo setting, they provide useful information on the comparative capacity of the tested probiotics in vitro to inhibit pathogen growth, adhere to host epithelial cells, and modulate host responses to common pathogens. Under the test conditions, the *B. velezensis* consortium in C1 CFS was more effective than the *B. licheniformis* and *B. subtilis* consortia in C2 and the probiotic *Clostridium butyricum* in C3 at inhibiting the growth of selected pathogenic isolates of ETEC. CFSs *C. perfringens* and *Salmonella* spp., derived from C1, were also more effective than CFSs from C2 or C3 at enhancing the TJ protein expression of ZO-1, MUC13, MUC20, and GAP junction protein and the expression of cell signaling markers betacellulin in porcine intestinal epithelial cells, both of which are critical for maintaining barrier integrity. Additionally, C1 CFS increased the production of anti-inflammatory cytokines, reduced the production of pro-inflammatory cytokines by these porcine epithelial cells, and modulated cytokine and chemical mediator production by macrophage-like 3D4/21 cells. These results build upon and reinforce our earlier in vitro findings with individual strains, providing mechanistic evidence that helps explain the beneficial outcomes reported in piglets supplemented with C1 in previous in vivo studies.

## Data Availability

The original contributions presented in this study are included in the article/Supplementary Materials. Further inquiries can be directed to the corresponding author.

## Supplementary Materials

The following supporting information can be downloaded at https://www.mdpi.com/article/10.3390/microorganisms14010249/s1, Table S1: Identification and genetic characterization of the Enterotoxigenic *Escherichia coli* (ETEC), *Clostridium perfringens*, and *Salmonella* isolates used in the assays; Table S2: Primers and Taq Man ID numbers used for PCR; Table S3: Percentage inhibition of individual pathogenic isolates by CFS derived from C1, C2, C3, and control strains.

## Abbreviations

The following abbreviations are used in this manuscript:

BHI	Brain heart infusion broth
CFU	Colony-forming units
CFS	Cell-free supernatant
CLECs	C-type lectin domain-containing proteins
ETEC	Enterotoxigenic *Escherichia coli*
FBS	Fetal bovine serum
FDR	False discovery rate
IPEC	Intestinal porcine epithelial cells
LB	Luria–Bertani broth
LCA	Latent class model
LT	Heat-labile toxin
MUC13	Mucin 13
MUC20	Mucin 20
OD	Optical density
P/S	Penicillin/streptomycin
PWD	Post-weaning diarrhea
RT-qPCR	Reverse-transcription quantitative polymerase chain reaction
SCFAs	Short-chain fatty acids
TEER	Transepithelial electrical resistance
TSA	Tryptic soy broth
ZO-1	Zonula occludens-1
