# Supplementary material for: A Probiotic Bacillus velezensis Consortium Exhibits Superior Efficacy over Two Alternative Probiotics in Suppressing Swine Pathogens and Modulating Intestinal Barrier Function and Immune Responses In Vitro
Source: Microorganisms. 2026 Jan 21;14(1):249. doi: 10.3390/microorganisms14010249 (PMC12843785; doi:10.3390/microorganisms14010249)
Supplement: Supplementary file 1 [file microorganisms-14-00249-s001.zip › microorganisms-4070861-supplementary.pdf]

Supplementary Table S1. Identification and genetic characterization of the Enterotoxigenic *Escherichia coli* (ETEC), *Clostridium perfringens* and *Salmonella* isolates used in the assays.

**IFF Collection Code**	**Genetic characterization (identified virulence markers)**
EC-2	F4/STa/STb
EC-3	F4/STb
EC-23	F18/STb/LT
EC-50	F18/STb
EC-58	F18/STx2e
EC-61	F18/STx2e
EC-62	F18/STx2e
EC-63	F18/STa/STb
EC-65	F18/STx2e
EC-87	Negative control
EC-88	F4/STb/LT
EC-89	F4/STb/LT
EC-90	Negative control
EC-91	Negative control
EC-93	Negative control
EC-94	Negative control
EC-95	Negative control
CpC9	C, ≤4 NE, cpb2+, netB-
CpC11	C, ≤4 NE, cpb2+, netB-
CpA3	A, ≤4 NE, cpb2-, netB-
CpA5	A, ≤4 NE, cpb2+, netB-
CpA10	A, 8-16 NE, cpb2-, netB-
CpA12	A, 8-16 NE, cpb2-, netB-
CpA15	A, 8-16 NE, cpb2+, netB-
CpA21	A, 32 NE, cpb2+, netB-
CpA23	A, ≥64 NE, cpb2-, netB-
CpA26	A, ≥64 NE, cpb2-, netB-
CpA29	A, ≥64 NE, cpb2+, netB-
Sal5	*Typhimurium*
Sal6	*Typhimurium*
Sal9	*Livingstone*
Sal10	*Livingstone*
Sal11	*Typhimurium*
Sal12	*Typhimurium*
Sal13	*Typhimurium*

Supplementary Table S2. Taq Man ID for PCR

**Genome target in PIEC-J2 cells**	**TaqMan ID No.**
IL-6	Ss03384604_u1
TGF-β	Ss04955543_m1
ZO-1	Ss03373514_m1
MUC13	Ss03386544_u1
MUC20	Ss03389869_m1
**House-keeping genes**	
Beta-Actin	Ss03376563_uH
HPRT	Ss03388274_m1

Supplementary Table S3. Percentage inhibition of individual pathogenic isolates by cell-free supernatant derived from probiotic C1, C2, C3, and control strains

Isolate code	*E. coli genotype*	C1A	C1B	C1C	C1	C2A	C2B	C2	C3	CC1	CC2A	CC2B	CC2	CC3
EC2	F4	64%	68%	64%	71%	7%	-2%	-2%	40%	-17%	26%	10%	7%	24%
EC3	F4	96%	88%	91%	91%	-11%	-10%	-16%	20%	-1%	-15%	-16%	-14%	35%
EC88	F4	81%	81%	81%	82%	-53%	-59%	-17%	-14%	-29%	1%	11%	1%	59%
EC89	F4	94%	97%	95%	95%	-7%	-13%	-3%	-5%	-2%	-7%	-8%	-8%	59%
EC23	F18	63%	55%	61%	63%	-23%	-15%	-17%	8%	-11%	-25%	-11%	-19%	30%
EC50	F18	89%	78%	82%	94%	-23%	-31%	-24%	-10%	-6%	-32%	-25%	14%	6%
EC58	F18	49%	49%	46%	47%	-30%	-18%	-13%	7%	3%	13%	11%	22%	20%
EC61	F18	67%	61%	61%	61%	-10%	-12%	-16%	9%	-2%	4%	15%	18%	38%
EC62	F18	62%	61%	60%	59%	7%	4%	-1%	34%	-8%	14%	20%	20%	27%
EC63	F18	46%	36%	39%	32%	-46%	-46%	-36%	-3%	0%	9%	-13%	-6%	30%
EC65	F18	69%	73%	65%	67%	-9%	-13%	-12%	14%	1%	0%	0%	-4%	3%
EC87	DN	58%	66%	55%	64%	-20%	-16%	-10%	4%	-7%	5%	17%	22%	32%
EC90	DN	97%	95%	94%	97%	-4%	5%	-1%	26%	3%	-4%	14%	17%	56%
EC91	DN	90%	91%	84%	88%	33%	23%	5%	-20%	-9%	-18%	17%	20%	44%
EC93	DN	73%	74%	68%	71%	-12%	-5%	-4%	9%	10%	-1%	19%	13%	22%
EC94	DN	73%	76%	60%	62%	-6%	-19%	-11%	25%	6%	-5%	13%	11%	22%
EC95	DN	63%	85%	52%	61%	-4%	7%	6%	4%	24%	1%	17%	14%	40%
Isolate code	CP genotype	C1A	C1B	C1C	C1	C2A	C2B	C2	C3	CC1	CC2A	CC2B	CC2	CC3
CpC9	C	99%	99%	100%	90%	10%	-3%	3%	7%	33%	4%	-3%	26%	-40%
CpC11	C	99%	99%	99%	99%	14%	20%	12%	6%	5%	10%	7%	63%	6%
CpA3	A	88%	89%	88%	88%	20%	9%	17%	12%	-4%	4%	58%	35%	86%
CpA5	A	96%	96%	96%	96%	12%	4%	15%	3%	100%	4%	-3%	30%	27%
CpA10	A	91%	90%	89%	91%	14%	4%	7%	10%	2%	8%	38%	9%	81%
CpA12	A	99%	97%	99%	98%	14%	4%	8%	14%	-10%	8%	3%	2%	95%
CpA15	A	100%	96%	90%	75%	4%	-2%	9%	0%	-3%	-1%	-15%	59%	27%
CpA21	A	100%	100%	100%	100%	6%	9%	3%	5%	28%	-2%	-4%	90%	8%
CpA23	A	93%	92%	91%	93%	12%	5%	7%	-3%	-11%	1%	-1%	3%	31%
CpA26	A	92%	87%	55%	58%	10%	4%	6%	-3%	-3%	-1%	-5%	1%	8%
CpA29	A	96%	95%	95%	96%	12%	6%	9%	-9%	31%	-9%	44%	41%	32%
Isolate code	*Salmonella genotype*	C1A	C1B	C1C	C1	C2A	C2B	C2	C3	CC1	CC2A	CC2B	CC2	CC3
Sal5	Try	68%	60%	60%	62%	-2%	-3%	-6%	13%	-8%	7%	4%	-14%	16%
Sal6	Try	56%	55%	50%	52%	-7%	0%	-12%	16%	-3%	5%	-10%	5%	22%
Sal11	Try	51%	47%	44%	42%	-15%	-13%	-12%	-2%	11%	-8%	-13%	-17%	9%
Sal12	Try	50%	44%	48%	48%	-12%	-10%	-2%	6%	13%	-7%	-7%	-16%	4%
Sal13	Try	61%		55%	57%	7%	-16%	-10%	23%	14%	21%	2%	0%	17%
Sal9	living	52%		53%	53%	12%	1%	-2%	20%	-11%	18%	6%	2%	19%
Sal10	living	46%		50%	51%	-1%	2%	-7%	-6%	-1%	-5%	-12%	-13%	9%
